# Effect of high-fat diet on the lipid profile of ovarian granulosa cells and female reproduction in mice

**DOI:** 10.1371/journal.pone.0287534

**Published:** 2023-06-27

**Authors:** Jinchun Gao, Mingchao Liu, Jingge Liu, Peihua Shi, Haoliang Cui, Shunran Zhao, Xinbo Zhang, Chenyu Tao

**Affiliations:** 1 College of Animal Science and Technology, Hebei Agricultural University, Baoding, Hebei Province, China; 2 College of Veterinary Medicine, Hebei Agricultural University, Baoding, Hebei Province, China; 3 College of Animal Science and Food Engineering, Jinling Institute of Technology, Nanjing, China; Max Delbruck Centrum fur Molekulare Medizin Berlin Buch, GERMANY

## Abstract

Currently, comorbidities of obesity are becoming increasingly frequent. For example, obese women are more susceptible to reproductive diseases; however, the underlying mechanism remains poorly understood. The present study aimed to explore the effect of obesity on female reproduction and discuss changes of the lipid profile in ovarian granulosa cells. Fifty female mice were randomly divided into two groups, one group was fed high-fat diet, the other group was fed standard control diet, food and water freely. After 12 weeks of feeding, the average body weight of the high-fat diet mice (19.027g) was significantly higher than that of the standard control diet mice (36.877g) (P < 0.05). The tissue sections were stained with oil red O, and the online software *mage Pro plus 6*.*0* analyzed the staining results, the lipids in the ovaries and endometria were found to be different between the two groups. Liquid chromatography-electrospray ionization with tandem mass spectrometry (LC-ESI-MS/MS) analysis of ovarian granulosa cells (GCs) was performed, with a total of 228 different lipids being identified, the abundant of 147 were increased and 81 were decreased in the high-fat diet group. Among them, PI (18:1/20:1) was the most different lipid, and high-fat feeding was 85 times higher than standard control group. Among these different lipids, 44% in phospholipid metabolism, 30% in glycerolipid metabolism, and 30% in fat digestion and absorption. The results of this study laid a theoretical foundation of the effects of diet-induced obesity on female reproduction.

## 1. Introduction

Currently, an increasing number of people suffer from obesity due to increased intake of calories and fat. Moreover, additional health problems caused by obesity have also become increasingly frequent, as obesity is correlated with an increased risk of hypertension, hyperlipidemia, heart disease, and even stroke [1]. In addition, obesity in females has been associated with infertility, with obese women being more susceptible to the development of reproductive diseases, including menstrual dysfunction, anovulation, and miscarriage. Obesity is common among women at reproductive age, with 38.3% of chinese women over the age of 20 years being classified as obese; therefore, obesity-associated reproductive dysfunction represents a major societal problem [2].

Investigations on the impact of a high-fat diet (HFD) on follicular development in the ovary revealed a reduced number of primordial follicles [3]. Furthermore, HFD exposure has been associated with follicular atresia. However, the effects of HFDs on different stages of follicles and corpora lutea are inconsistent, with studies describing variable changes in the different follicle pools [4]. Maternal obesity can also impair embryo development and offspring health [5–7]. Stella (also known as DPPA3 or PGC7) protein in oocytes was significantly decreased in HFD-based female mouse, leading to accumulation of maternal 5-hydroxymethylcytosine modifications and DNA lesions, which may be a critical mechanism that mediates the phenotypic effects of maternal obesity in embryos and offspring [8]. Maternal obesity has been associated with shifts in intestinal microbiota, which may contribute to impaired barrier function, thus, leading the placenta and fetus to pro-inflammatory mediators. Placenta from diet-induced obese mothers showed blood vessel immaturity, hypoxia, increased transcript levels of inflammation, autophagy and altered levels of endoplasmic reticulum stress markers [9].

The lipids which were increased in abundance in the ovary have also been observed in response to HFDs [10–12]. Increased numbers of lipid droplets in the ovaries were observed in rats maintained on a HFD with approximately 60% lipids for 180 days [13]. Moreover, increased lipid content and abnormal lipid accumulation patterns were observed in germinal vesicle oocytes of C57BL/6J mice after fed a 60% HFD for 6 weeks [14]. Additionally, lipid accumulation increased in both cumulus cells and oocytes of CBA mice that were fed a 40% HFD for 4 weeks. This was also associated with increased lipotoxicity with concomitant increased markers of endoplasmic reticulum stress, decreased mitochondrial membrane potential, and increased apoptosis, along with increased rates of an ovulation [15].

In this study, a total of 50 mice were fed a high-fat diet with 60% fat or standard control diet of 15% fat for 12 weeks, after which the reproductive organs, such as the ovaries, uterus, and fallopian tubes, were observed by oil red O staining. According to oil red O staining data, the lipids in the ovaries were found to be different between the two groups. The ovarian GCs are the most abundant cells in the ovary and are crucial for oocyte development. Thus, we hypothesized that the change of lipid profiles in ovary GCs altered the metabolic pathways, and this may cause some reproductive problems even influence the quality of oocytes. To verify this hypothesis, first, establishment of a mouse obesity model induced by a high-fat diet, and the mice were adapted to the obesity state. To evaluate the effects of DIO (diet-induced obesity) on ovarian lipid composition, to understand if pathways related to fertility or ovarian follicle quality are altered and the lipidomics analysis of ovarian GCs were conducted. This study aimed to assess the effect of a high-fat diet on ovarian lipid composition to see if pathways related to fertility or follicle quality are altered and evaluate the effects of diet-induced obesity on female reproduction.

## 2. Materials and methods

### 2.1 Animals and feed

All experiments were performed in accordance with the Guide for the Care and Use of Laboratory Animals of Hebei Agricultural University, Baoding, China. The protocol was approved by the Institutional Animal Care and Use Committee of Hebei Agricultural University. All efforts were made to minimize animal suffering. The study is reported in accordance with ARRIVE guidelines.

All the test mice were purchased from Beijing Weitong Lihua Laboratory Animal Technology Co. The experimental animals were selected from Kunming mice. KM mice were the most productive and used outbred mice in China. It is characterized by strong disease resistance and adaptability, high reproductive rate and survival rate. Four-week-old of female Kunming mice (n = 50) weighing approximately 15 ± 2 g were housed at 23 ± 2°C with 55 ± 5% relative humidity, and in a 12 h light/dark cycle. All mice were fed with standard feed for 1 week before the start of the experiment. The mice were then separated and fed standard control diet of 15% fat (CON group; n = 25) or a high-fat diet with 60% fat (HFD group; n = 25). The feed is purchased from Xiaoshu Youtai Beijing Biotechnology Co., LTD. The ingredient composition of the standard feed is shown in S1 and S2 Tables. The obesity model was established successfully when the weight of the HFD group stabilized by more than 20% of the CON group. The experiment was performed for 3 months, during which the body weights of the mice were recorded weekly. The animals were given a continuous supply of drinking water and feed. In all cages, drinking bottles and paddings were disinfected under high pressure. At the end of the experiment, all mice were euthanized.

### 2.2 Collection and culture of mouse oocyte

After the obesity model was established, five mice in each HFD group and CON group were randomly selected and given 10 IU pregnant mare’s serum gonadotropin (PMSG) for hyperovulation. Forty-eight hours after pmsg, use a scalpel to cut the skin from the back of the mouse along the spine, find the ovary, use surgical tweezers to break the film, and remove the ovary, Transfer to M2 medium preheated at 37°C (Sigma), the ovaries were sliced and squeezed with a 1 mL syringe, and oocytes were released. Under the ocular lens of a 2-fold objective microscope, the germinal vesicle stage oocytes (GV oocytes) with light color, clear outline, round shape and few granulocytes were moved to the drops of M2 medium with a capillary glass tube, and the granulocytes on the oocytes were gently blown and vacuumed with a capillary glass tube to remove the damaged cells. More than 100 GV oocytes were collected from each group. The GV oocytes were obtained and cultured at 38.5°C and 5% CO_2_ in air, at maximum humidity for 12 hours. The extrusion of polar body 1 (PB1) was observed under the microscope.

### 2.3 Data collection of total litter size

After the successful establishment of the obesity model, twenty healthy male mice of the same breed were prepared for mating experiments. Firstly, estrus identification was performed on female mice. The tail of the female mouse was raised to expose the genitalia, and the external genitalia was opened with surgical forceps. If redness or swelling was found, it was considered estrus. Then each female mouse in estrus was paired with a male mouse and placed in a cage, if a vaginal plug formed by white semen is observed in the vulva the next day, it is considered successful mating, otherwise wait. The mice maintained their previous diet during mating and pregnancy. All 10 mice in each group were successfully mated and all gave birth to litters. This indicates that for first conception there is no difference between obese and normal mice in their ability to fertilize and become pregnant. Finally, observe the mice daily and promptly record the number of offspring produced.

### 2.4 Glucose and hepatic triglyceride testing

At the end of superovulation and mating selection, the remaining 10 mice in each group fasted all night, and euthanized with isoflurane /CO2 anesthesia to prevent excessive hormone level fluctuations caused by stress. Immediately collect blood samples from the inferior vena cava when the mice lose mobility, and immediately test the blood samples for glucose and liver triglyceride levels. Glucose and hepatic triglyceride levels were analyzed using the electrode method (Glutest Neo Super; Sanwa Kagaku Kenkyusho, Aichi, Japan).

### 2.5 Oil red O staining

Briefly, randomly select three mice from each group in the hormone detection group and remove ovarian tissue, the tissue block is placed flat in a flexible plastic bottle cap or a special box (diameter about 2 cm). If the tissue block is small enough to absorb the tissue with OCT embedding agent, then the special box is slowly placed flat into a small cup containing liquid nitrogen. At 10~20 s, the ice quickly forms a block. Take out the tissue ice cubes and put them in the -80°C refrigerator for reserve or in the constant cold box microtome for frozen section with the thickness of 8~15 μm. Rewarm and dry the frozen slices, fix them for 15 minutes, rinse with running water, and let dry. tissue slices were covered with a few drops of oil red O stain (diluted 1:0.6 with distilled water) for 5 min. The slides were then rinsed under running tap water and counterstained by immersion for 5 min in Mayer’s hematoxylin (Diapath Microstain, Martinengo, Italy). After rinsing again under running tap water and air drying, the slides were covered with a drop of aqueous mounting fluid (Crystal Mount; Biomeda, Foster City, CA, USA), then covered with balsam (Eukitt; Electron Microscopy Sciences, Hatfield, PA, USA) and a coverslip. The slices were scanned using a digital scanning system, which could be magnified up to 500 times for observation and statistics, and the results were evaluated using online software *Image Pro plus 6*.*0*.

### 2.6 Lipidomics analysis of ovarian granulosa cells

#### Chemicals and reagents

High-performance liquid chromatography (HPLC)-grade acetonitrile, methanol, isopropanol, and tert-butyl methyl ether were purchased from Merck (Darmstadt, Germany). HPLC-grade formic acid was purchased from Sigma-Aldrich (St. Louis, MO, USA). Dichloromethane and ammonium formate were purchased from Thermo Fisher Scientific (Waltham, MA, USA). Ultrapure water was obtained using a Milli-Q system (Millipore, Billerica, MA, USA). Lipid standards were purchased from Sigma-Aldrich or Avanti Polar Lipids (Alabaster, AL, USA).

#### Sample preparation and extraction

Samples were placed in liquid nitrogen for 2 min, then thawed on ice for 5 min. The first step begins with the vortex treatment, repeating three times for 1 min each time. Each sample was centrifuged at 5000 rpm at 4°C for 1 min, homogenized in 1 mL of methanol, tert-butyl methyl ether, and internal standard mixture, and stirred for 15 min. Then, 200 μL of water was added, and the mixture was stirred for 1 min and centrifuged at 12 000 rpm at 4°C for 10 min. A total of 500 μL of the supernatant was collected and concentrated. Samples were reconstituted in 200 ul of mobile phase B, and then stored at −80°C. Finally, the dissolving solution was placed in a sample bottle for liquid chromatography-electrospray ionization with tandem mass spectrometry (LC-ESI-MS/MS) analysis.

#### HPLC conditions

The sample extracts were analyzed using an LC-ESI-MS/MS system (ExionLC AD equipped with a QTRAP System; Sciex, Framingham, MA, USA). The analytical conditions were as follows: column, Thermo Accucore C30 (2.6 μm, 2.1 mm × 100 mm; solvent system A: acetonitrile/water [60/40 (v/v), 0.1% formic acid, 10 mmol/L ammonium formate], and B: acetonitrile/isopropanol [10/90 (v/v), 0.1% formic acid, 10 mmol/L ammonium formate]); gradient program A/B (v/v), 80:20 at 0 min, 70:30 at 2 min, 40:60 at 4 min, 15:85 at 9 min, 10:90 at 14 min, 5:95 at 15.5 min, 5:95 at 17.3 min, 80:20 at 17.3 min, and 80:20 at 20 min; flow rate, 0.35 mL/min; temperature, 45°C; and injection volume, 2 μL. The effluent was alternatively connected to an ESI-triple quadrupole linear ion trap (QTRAP)-MS.

#### ESI-QTRAP-MS/MS conditions

Linear ion trap and triple quadrupole (QQQ) scans were acquired using a QTRAP LC-MS/MS System equipped with an ESI Turbo Ion-Spray interface, operating in positive and negative ion mode, and controlled by Analyst 1.6.3 software (Sciex). The ESI source operation parameters were as follows: ion source, turbo spray; source temperature, 500°C; ion spray voltage, 5500 V (positive) and −4500 V (negative); ion source gas 1, gas 2, and curtain gas were set at 45, 55, and 35 psi, respectively; and the collision gas was medium. Instrument tuning and mass calibration were performed with 10 and 100 μmol/L polypropylene glycol solutions in the QQQ and linear ion trap modes, respectively. QQQ scans were acquired as multiple reaction monitoring (MRM) experiments with a collision gas (nitrogen) set at 5 psi. Declustering potential and collision energy for individual MRM transitions were performed with further optimization. A specific set of MRM transitions was monitored for each period, according to the metabolites eluted within the period.

### 2.7 Availability of data

The datasets used and analysed during the current study available from the corresponding author on reasonable request.

### 2.8 Statistical analysis

Body weight data were analyzed using the MIXED procedure of SAS (SAS Inst. Inc., Cary, NC, USA); the model included treatment, time, and interaction as fixed effects, and replicates of individual effect as random effects. The PDIFF option adjusted by the Tukey method was included in the LSMEANS statement to account for multiple comparisons among treatments. Differences between treatments were declared significant at P ≤ 0.05.

Unsupervised principal component analysis of lipidome data was performed using the statistical function prcomp in R (www.r-project.org). The data were unit variance scaled before being analyzed.

Hierarchical cluster analysis of samples and metabolites are presented as heatmaps with dendrograms, with normalized signal intensities of the metabolites (unit variance scaling) being visualized as a color spectrum. Pearson correlation coefficients between samples were calculated using the cor function in R and presented as heatmaps. Both analyses were performed using the R package ComplexHeatmap.

Significantly regulated metabolites between groups were determined by variable importance in project ≥ 1 and absolute Log2FoldChange ≥ 1. Variable importance in project values were extracted from the orthogonal projections to latent structures discriminant analysis (OPLS-DA) data, which also contained score and permutation plots generated using the R package MetaboAnalystR. The data were log transformed (log2) and mean-centered before OPLS-DA. To avoid overfitting, a permutation test (200 permutations) was performed.

Identified metabolites were annotated using the Kyoto Encyclopedia of Genes and Genomes (KEGG) Compound database (http://www.kegg.jp/kegg/compound/), and annotated metabolites were mapped to the KEGG Pathway database (http://www.kegg.jp/kegg/pathway.html) [16]. Pathways associated with significantly regulated metabolites were then further assessed by metabolite set enrichment analysis, and their significance was determined using hypergeometric tests. P-values < 0.05 were deemed statistically significant.

## 3. Results

### 3.1 Changes in reproductive organs between HFD and CON mice

**Fig 1:**
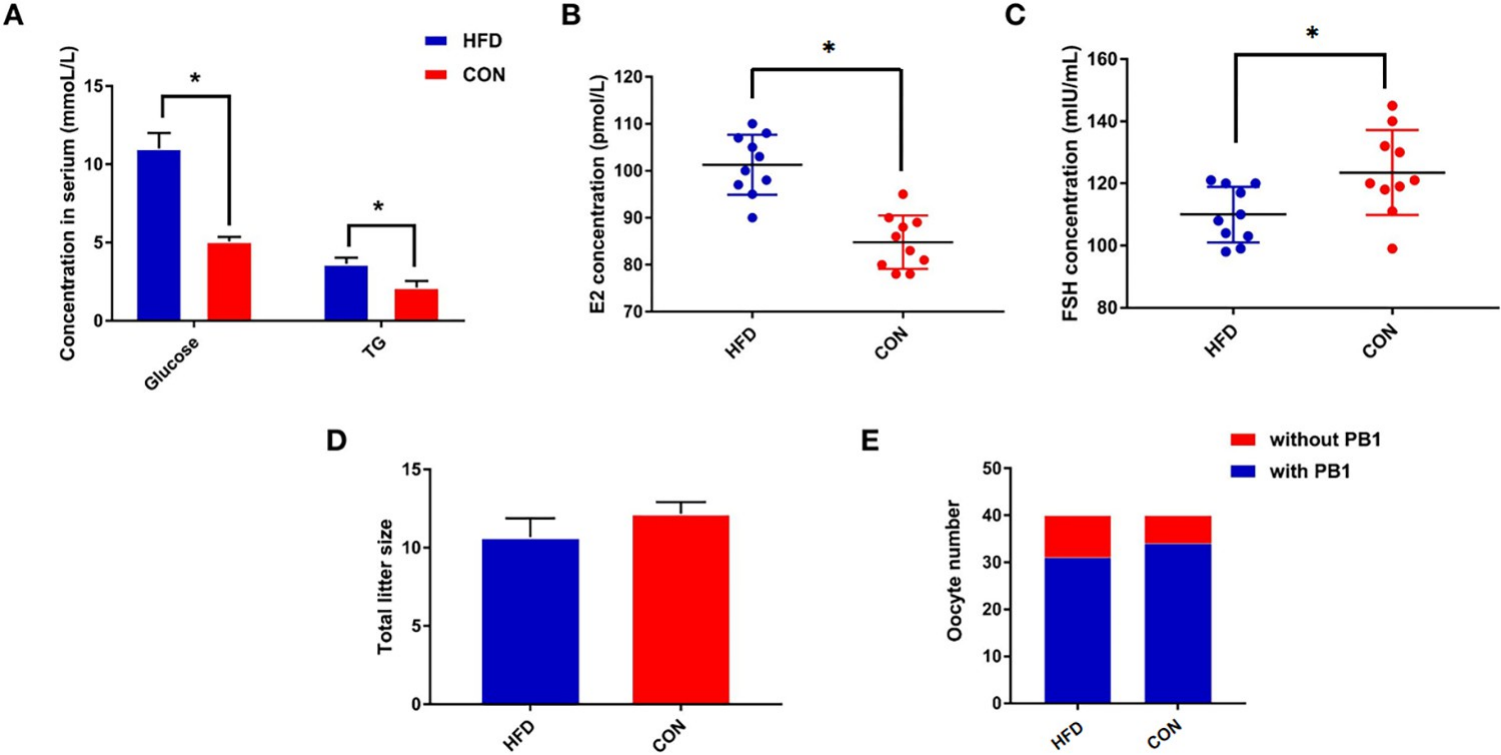
Changes in hormone and reproduction between HFD and CON mice. Effects of sustained high fat diet induction on blood hormone levels (N  =  10). (A) comparison of serum glucose and hepatic triglyceride concentrations between the two groups at week 12,and (B) Comparison of serum E2 concentration between the two groups at week 12,and (C) Comparison of serum FSH concentration between the two groups at week 12. (D) Comparison of total litter size between the two groups (N  =  10). (E) Comparison of PB1 count between the two groups (N  =  5). Values are presented as mean + S.E.M.. Significant differences following one-way ANOVA: *P < 0.05.

After the 12-week experimental period, the body weights differed significantly between groups (S1A and S1B Fig). Both the serum glucose and hepatic triglyceride levels were significantly higher in the HFD group than in the CON group (Fig 1A), indicating successful establishment of the obesity model of mice. The circulating level of E2 was higher in HFD group (P<0.05, Fig 1B), and FSH levels were lower in HFD versus CON mice. (P<0.05, Fig 1C). The litter size of the mice between two groups were also calculated, which Birth litter size was not different between the groups. (er in HFD group (P<0.05, Fig 1B), and FSH levels were lower in HFD versus CON mice. (P>0.05, Fig 1D). GV oocytes were collected and the extrusion of PB1 was slightly decreased in HFD mice as compared to those in the ND group (Fig 1E).

**Fig 2:**
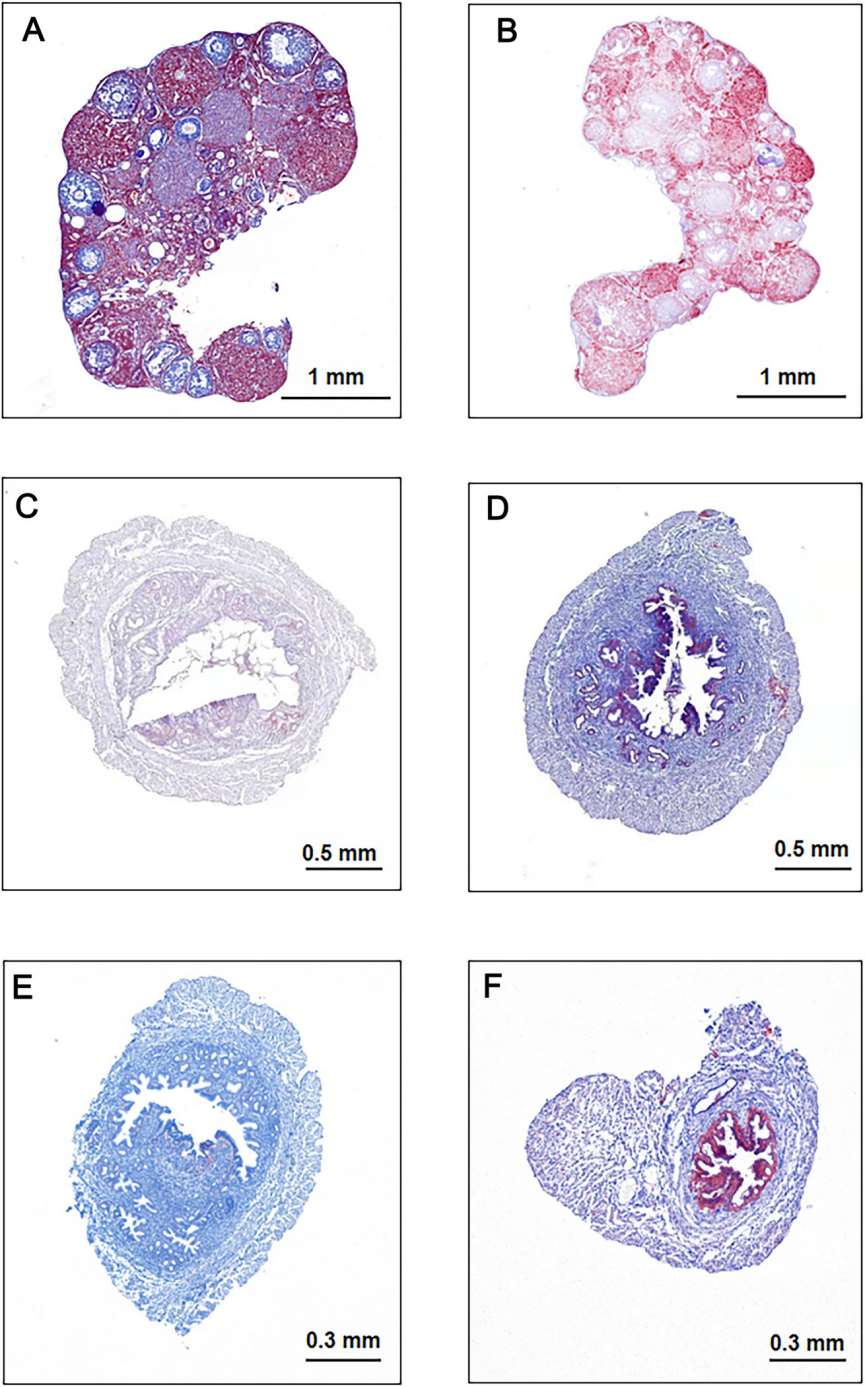
Oil red O staining of the reproductive organs of high-fed diet (HFD) and standard diet (CON) mice. (A) Oil red O staining of ovaries in CON mice. (B) Oil red O staining of ovaries in HFD mice. (C) Oil red O staining of uteri in CON mice. (D) Oil red O staining of uteri in HFD mice. (E) Oil red O staining of fallopian tubes in CON mice. (F) Oil red O staining of fallopian tubes in HFD mice.

Next, the reproductive organs of mice were stained with oil red O. The lipid content in the ovaries of HFD-fed mice was increased as compared with that of mice in the CON group (Fig 2A and 2B, P<0.5). Our results also showed that the lipid content in the endometria was increased (Fig 2C and 2D, P<0.5). However, no significant difference was observed between the amount of lipids in the uteri and fallopian tubes (Fig 2E and 2F).

### 3.2 Variance analysis of the lipid changes in GCs between HFD and CON mice

**Fig 3:**
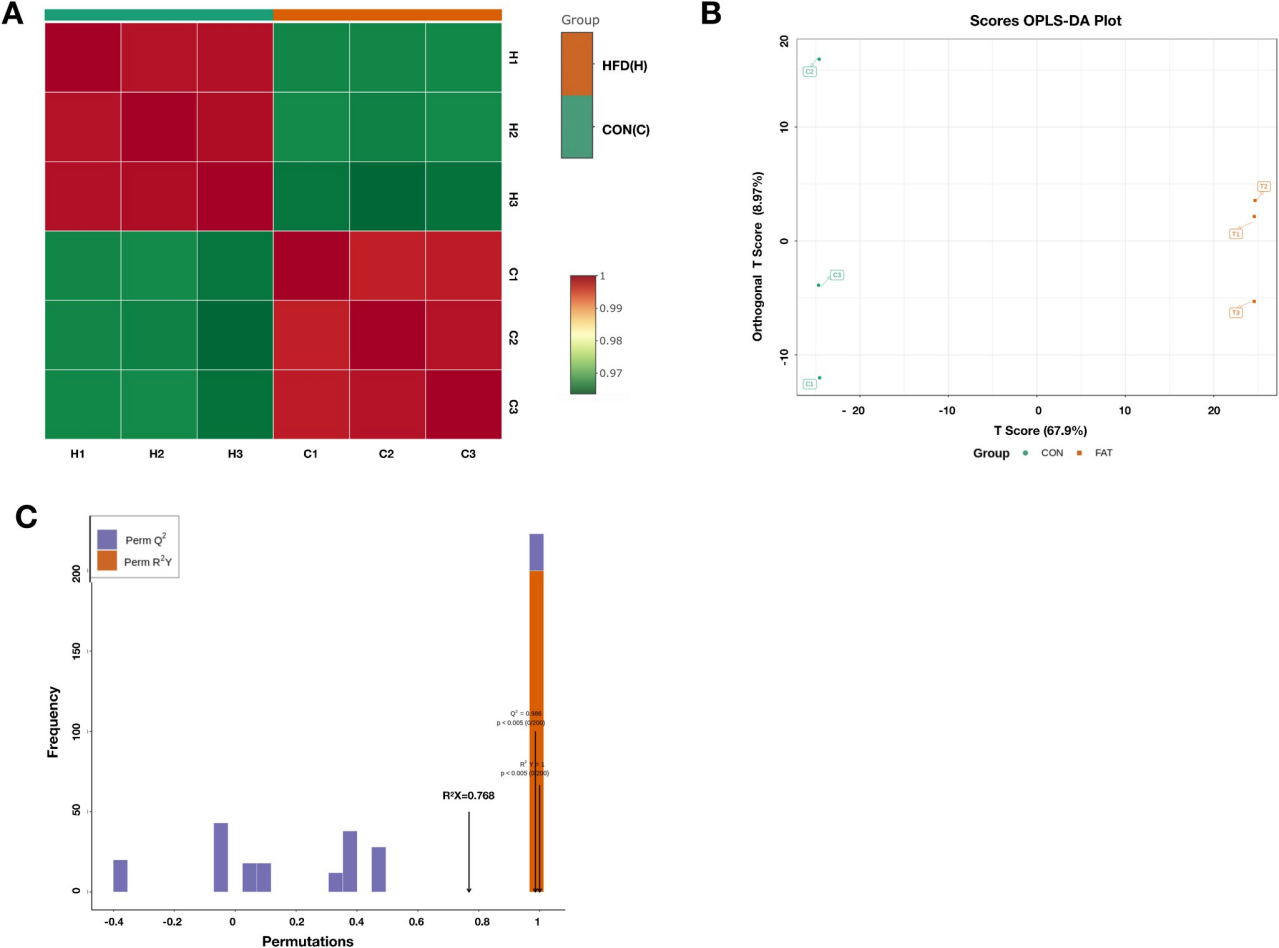
Correlation and difference analysis between ovarian granulosa cells in high-fed diet (HFD) and standard diet (CON) mice. (A) Heatmap of the Pearson’s correlation between HFD and CON groups. (B) Orthogonal projections to latent structures discriminant analysis (OPLS-DA) score plot of the lipids in ovarian granulosa cells from the HFD and CON groups. Profiles of HFD (green dots) and CON (red dots) are separated. (C) CON vs. HFD OPLS-DA permutation. The abscissa represents the accuracy of the model, and the ordinate represents the frequency of the model classification effect.

Since the lipid content in the ovaries differed between the two groups, the lipidomics of ovarian granulosa cells was conducted to reveal the crucial lipids accumulated in the obese female reproductive system. All the euthanized mice were dissected, and all ovaries were collected. Three replicates were set in both the high-fat group and the control group, and granulosa cells of ovarian follicles were collected. The correlation and differences between the two treatment groups were calculated. Correlation analysis between the samples showed that the two groups were significantly different (r = 0.96), whereas the Pearson correlation coefficient between samples within the same group was approximately 0.99 (Fig 3A). OPLS-DA was used as a supervised multivariate data analysis method to analyze the lipids between the HFD and control groups. Based on the lipid dataset analyses, the profiles in the HFD group were distinct from those in the control group (Fig 3B). The accuracy of the model was verified (Fig 3C) based on 200 random permutation and combination experiments conducted on the data, with Q2 = 0.986 (P < 0.005) and R2Y = 1 (P < 0.005), indicating that the model was optimal.

### 3.3 Analysis of the lipid landscape

**Fig 4:**
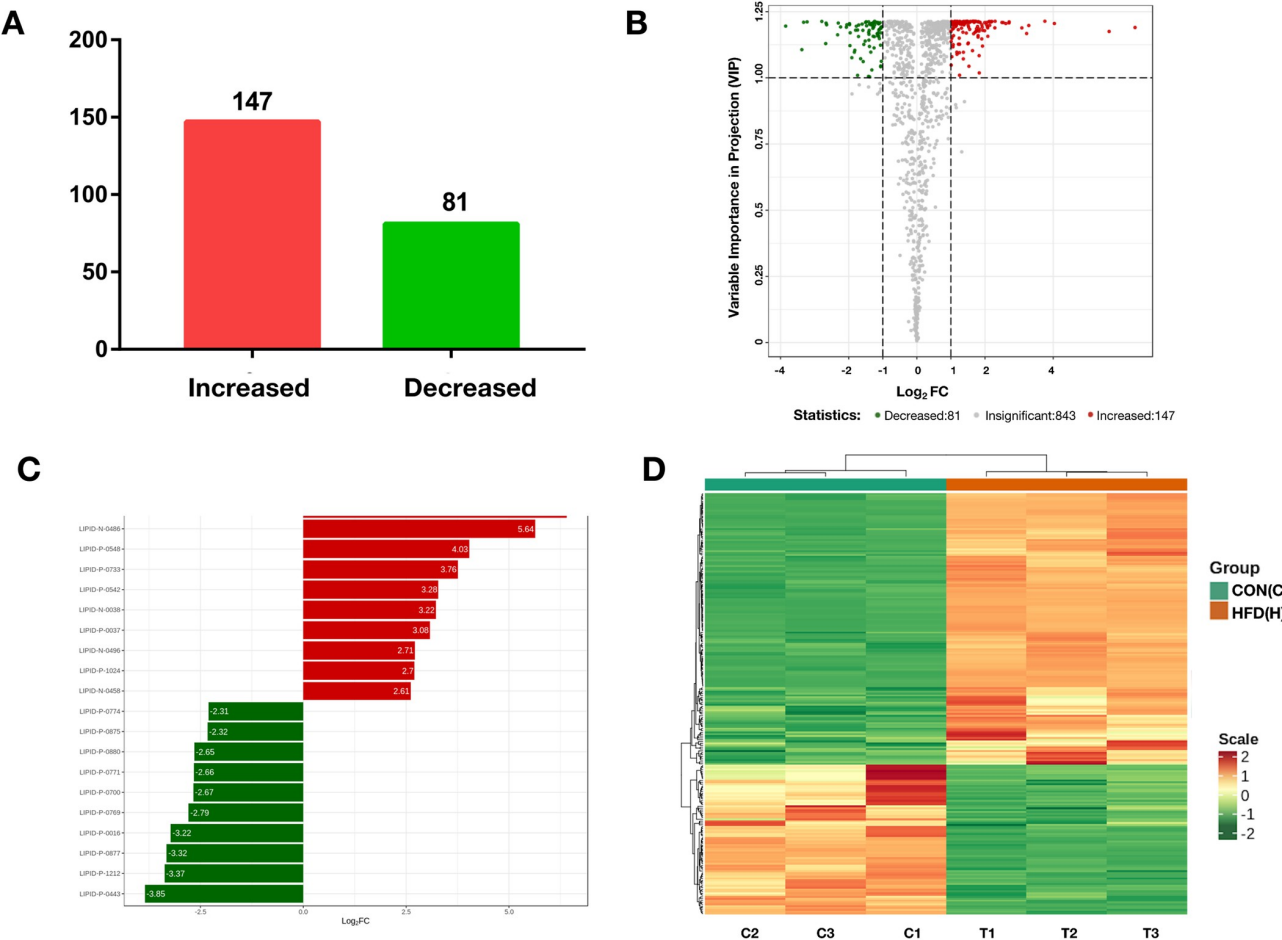
Identification of different lipids in ovarian granulosa cells between mice in high-fed diet (HFD) and standard diet (CON) groups. (A) Number of different lipids. Red and green represent increased and decreased lipids, respectively. (B) Volcano plot of the different lipids. The red dot represents 2-fold (right) and 0.5-fold (left) variation and P < 0.05. A total 228 of lipids with significant changes were identified based on differences between the two groups. (C) Histogram of the fold-change values of the top different lipids. Red and green represent increased and decreased lipids, respectively. (D) Heatmap of different lipids. The color is proportional to the intensity of the changes. Red and green represent increased and decreased lipids, respectively.

**Fig 5:**
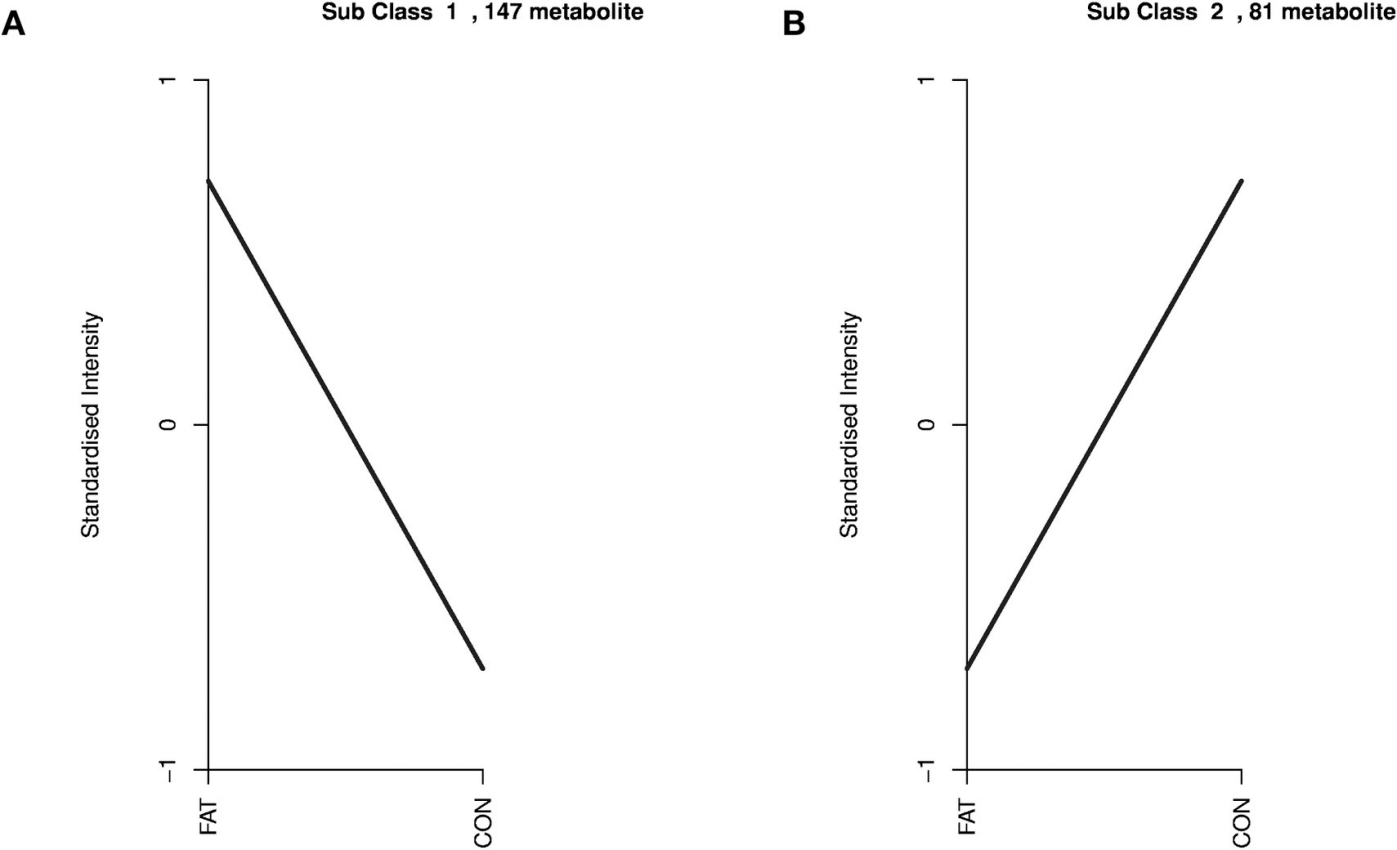
K-means analysis of the different lipids of increased (A) and decreased (B) lipids between the two groups. The abscissa represents the sample and the ordinate represents the normalized relative metabolite content.

According to the lipidomic results of the ovarian granulosa cells, a total of 228 lipids with significant differences were identified between the two groups, the abundant of 147 lipids were increased and 81 were decreased in the HFD group (Fig 4A and 4B). Among the top 10 different lipids (Fig 4C, S2 Fig and Table 1), PI (18:1/20:1) was the most different lipid, and was 85-fold higher in the HFD group than in the CON group. Lipid differences between the groups are shown in a clustering heatmap (Fig 4D) and K-means plot (Fig 5).

**Table 1:** Top 10 different lipids from the lipidomic analysis of ovarian granulosa cells.

Formula	Compounds	Class I	Class II	Fold_Change	Type
C47H87O13P	PI(18:1/20:1)	GP glycerophospholipids	PI Phosphatidyl inositol	85	increased
C47H89O13P	PI(18:0/20:1)	GP glycerophospholipids	PI Phosphatidyl inositol	50	increased
C50H98NO7P	PC(O-18:1/24:1)	GP glycerophospholipids	PC-O Phosphatidylcholie	16	increased
C43H89N2O6P	SM(d18:0/20:0)	SL sphingolipid	SM sphingomyelin	14	increased
C50H100NO7P	PC(O-18:0/24:1)	GP glycerophospholipids	PC-O phosphatidylcholie	10	increased
C20H32O3	(±)18-HETE	FA Fatty acyl	Eicosanoid oxidized lipid	9	increased
C21H39NO4	Carnitine C14:1	FA Fatty acyl	CAR Acylcarnitine	8	increased
C45H83O13P	PI(18:1/18:1)	GP glycerophospholipids	PI Phosphatidyl inositol	7	increased
C65H120O6	TG(18:1/20:1/24:1)	GL glycolides	TG triacylglycerol	7	increased
C42H73O10P	PG(16:1/20:4)	GP glycerophospholipids	PG phosphatidyl glycerol	6	increased
C21H42NO6P	LPE(P-16:1)	GP glycerophospholipids	LPE-P Lysophosphatidyle-thanolamine	0.07	decreased
C57H92O6	TG(18:2/18:3/18:4)	GL glycolides	TG triacylglycerol	0.10	decreased
C43H78O6	TG(10:0/12:0/18:2)	GL glycolides	TG triacylglycerol	0.10	decreased
C19H37NO5	Carnitine C12-OH	FA Fatty acyl	CAR Acylcarnitine	0.11	decreased
C39H74O6	TG(10:0/12:0/14:0)	GL glycolides	TG triacylglycerol	0.14	decreased
C43H82NO10P	PS(18:1/19:0)	GP glycerophospholipids	PS phosphatidylserine	0.16	decreased
C41H78O6	TG(10:0/16:0/12:0)	GL glycolides	TG triacylglycerol	0.16	decreased
C45H82O6	TG(8:0/16:0/18:2)	GL glycolides	TG triacylglycerol	0.16	decreased
C43H78O6	TG(8:0/14:0/18:2)	GL glycolides	TG triacylglycerol	0.20	decreased
C43H82O6	TG(10:0/14:0/16:0)	GL glycolides	TG triacylglycerol	0.20	decreased

### 3.4 KEGG analysis of different lipids

**Fig 6:**
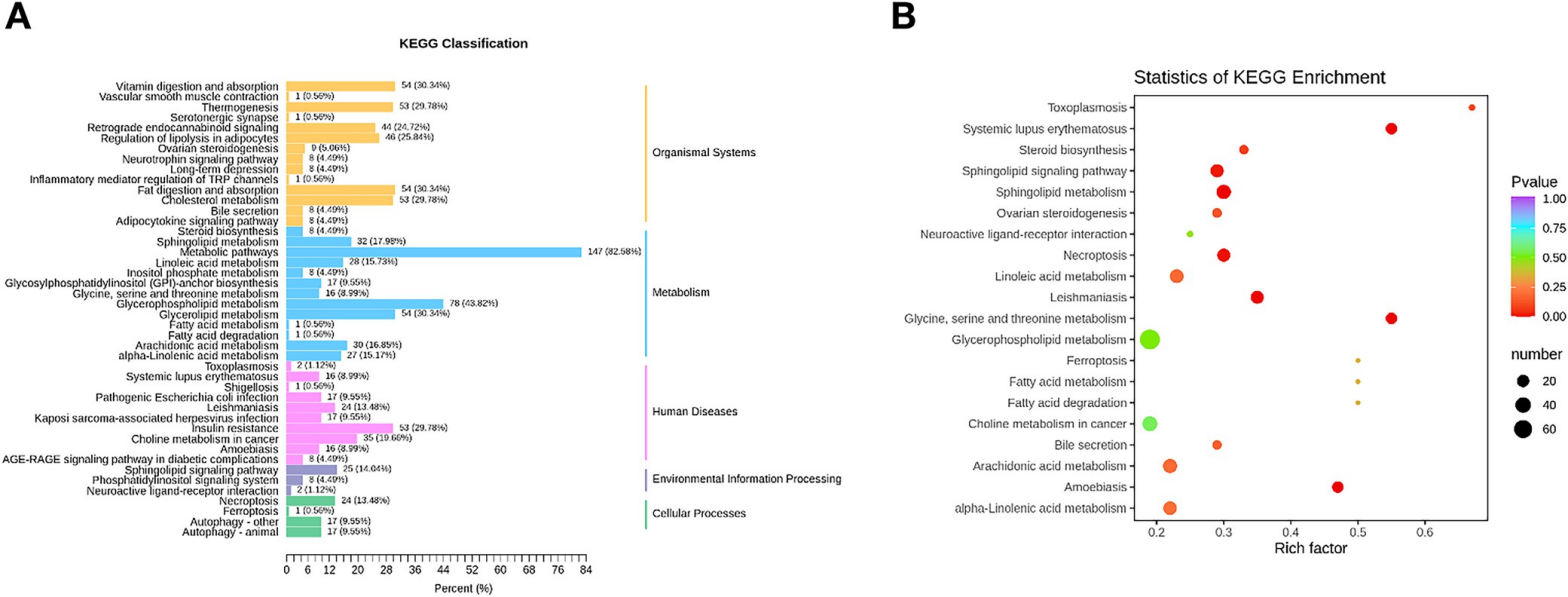
Kyoto Encyclopedia of Genes and Genomes (KEGG) analysis of the different lipids of high-fed diet (HFD) and standard diet (CON) groups. (A) KEGG classification barplot of different lipids. (B) KEGG pathway enrichment analysis of the identified different lipids. The rich factor represents the ratio of the number of different lipids in the corresponding pathway to the total number of lipids detected and annotated in the pathway. The size of the dots represents the number of metabolites enriched in the corresponding pathways with significant differences.

The annotated results according to KEGG compounds with significant differences between HFD and CON groups were further classified by pathway type (Fig 6A). Among the different lipids, a total of 147 (83%) were found to be involved in metabolic pathways, followed by 78 (44%) in phospholipid metabolism, 54 (30%) in glycerolipid metabolism, and 54 (30%) in fat digestion and absorption (Fig 6B). Overall, the different lipids were involved in glycine, serine, threonine, and phospholipid metabolism.

## 4. Discussion

According to the World Health Organization in 2014, there were 1.9 billion overweight adults worldwide, of which 600 million were obese [17]. Thus, obesity is a global health concern as it represents a serious risk factor for numerous chronic diseases. It is widely accepted that obesity results from prolonged positive energy balance. An increasing number of studies have focused on the mechanisms and effects of obesity.

Most studies on obesity have used mice as an experimental model because of their short reproductive cycle and low cost. However, the criteria for successful establishment of the obesity model have differed among studies. Some have shown that it takes 7–9 months to ensure that the body weight of the mice changes significantly [18]. There are several standards for the establishment of high-fat diet mice, for example, the body weight of mice in HFD group are 20% higher than that of CON group. It is also adopted in some studies that the body weight of mice in HFD group are significantly higher than that of CON group [19, 20]. In this study, the difference in body weight was significant at week 3 (P < 0.05), with a difference of up to 20%. Nevertheless, the mice were continuously fed for 12 weeks.

In the present study, during the feeding process, the mice in the HFD group showed symptoms of anorexia with a concomitant slight decrease in body weight at week 5. However, anorexia subsided a few days after. During this period, we paid close attention to the breeding environment and water intake of the mice to guarantee the good health of the animals.

The obesity models on female reproduction was quite complex, because of the complexities of the female reproductive system, which involves multiple organs working in conjunction with other systems, including the endocrine system [21]. Thus, isolating the features of the reproductive system can be a challenge. As demonstrated by the results of the present study, there were no differences in litter size and ovulation between the HFD and CON groups. This murine obesity model did not cause a difference in litter size, possibly because the weight difference was not large enough. In humans, obesity is associated with an increased risk of infertility in women. However, in this mouse obesity model, the rapid weight increase over a 3-month period was not enough to make a difference in ovulation and litter size. However, the maturation of GV oocytes in HFD groups was significantly decreased compared to CON group. The experiment result showed that the changes of lipids in ovary did influence the quality of oocytes. Ribeiro et al. conducted a systematic review and meta-analysis of the effects of obesity on female reproduction and found that overweight or obesity had only a weak negative impact on clinical pregnancy, live birth and miscarriage rates, while mature oocyte count and hormonal effects had a sustained effect [22]. Animal models of maternal obesity reveal that lipotoxicity in the ovarian environment is a key driver of oocyte defects, not only reducing developmental capacity, but also having long-term effects on offspring health [23]. Moreover, differences in lipids were only detectable in the ovaries through sectional observation of the reproductive organs, Granulosa cells, as the most important large population cells for ovarian follicle development and maturation, secrete androgens and estradiol, therefore, ovarian granulosa cells were selected as the research focus in the subsequent experiments.

Lipidomic analysis of ovarian granulosa cells showed a significant difference between the two groups, demonstrating that the amount and type of lipids in the ovarian granulosa cells of HFD-fed mice were significantly different from those in the CON group. Among them, 147 lipids were increased and 87 were decreased in the HFD group. Most of the different lipids were found to be phospholipids and glycerides. In particular, PI (18:1/20:1) abundance were found to be 84.58-fold higher in HFD-fed mice, whereas LPE (P-16:1) abundance were decreased to 0.07-fold compared to the CON group. Furthermore, the different lipids were found to be involved in metabolism-related pathways (83%), followed by phospholipid metabolism (44%) and glycerolipid metabolism (30%). Taken together, these findings suggest that different lipid metabolites may play an important role in lipid metabolism and thus regulate the function of granulosa cells in reproduction.

## 5. Conclusion

The lipids in the ovaries and endometria were found to be different between the high-fat diet and standard diet groups. Lipidomic analysis of ovarian granulosa cells showed that most of the different lipids were enriched in fat digestion, absorption, and metabolic pathways. As the most important nutritional support and hormone regulating cells of ovary, ovarian granulosa cells are crucial to the development and maturation of ovarian follicles, which are related to ovarian function. Therefore, we will focus on the influence of granulosa cell lipid metabolism disorders on ovarian aging in the future.

## Supporting information

S1 Fig Establishment of the obesity mouse model. (A) Continuous assessment of the body weights of the mice during the 12-week feeding period. Values are presented as mean + S.E.M. (N  =  25), “*” in the figure indicates a significant difference in body weight between the two groups of mice at this time. (B) Comparison of the high-fed diet (HFD) and standard diet (CON) mice at week 12.
(TIF)

S2 Fig Violin plot of the top ten differential lipid metabolism. (TIF)

S1 Table Ingredient composition and nutrient value of the standard feed. (PDF)

S2 Table Ingredient composition of high fat diet (HFD). (PDF)
