# Supplementary figures and images for: Effect of high-fat diet on the lipid profile of ovarian granulosa cells and female reproduction in mice
Source: PLoS One. 2023 Jun 27;18(6):e0287534. doi: 10.1371/journal.pone.0287534 (PMC10298767; doi:10.1371/journal.pone.0287534)

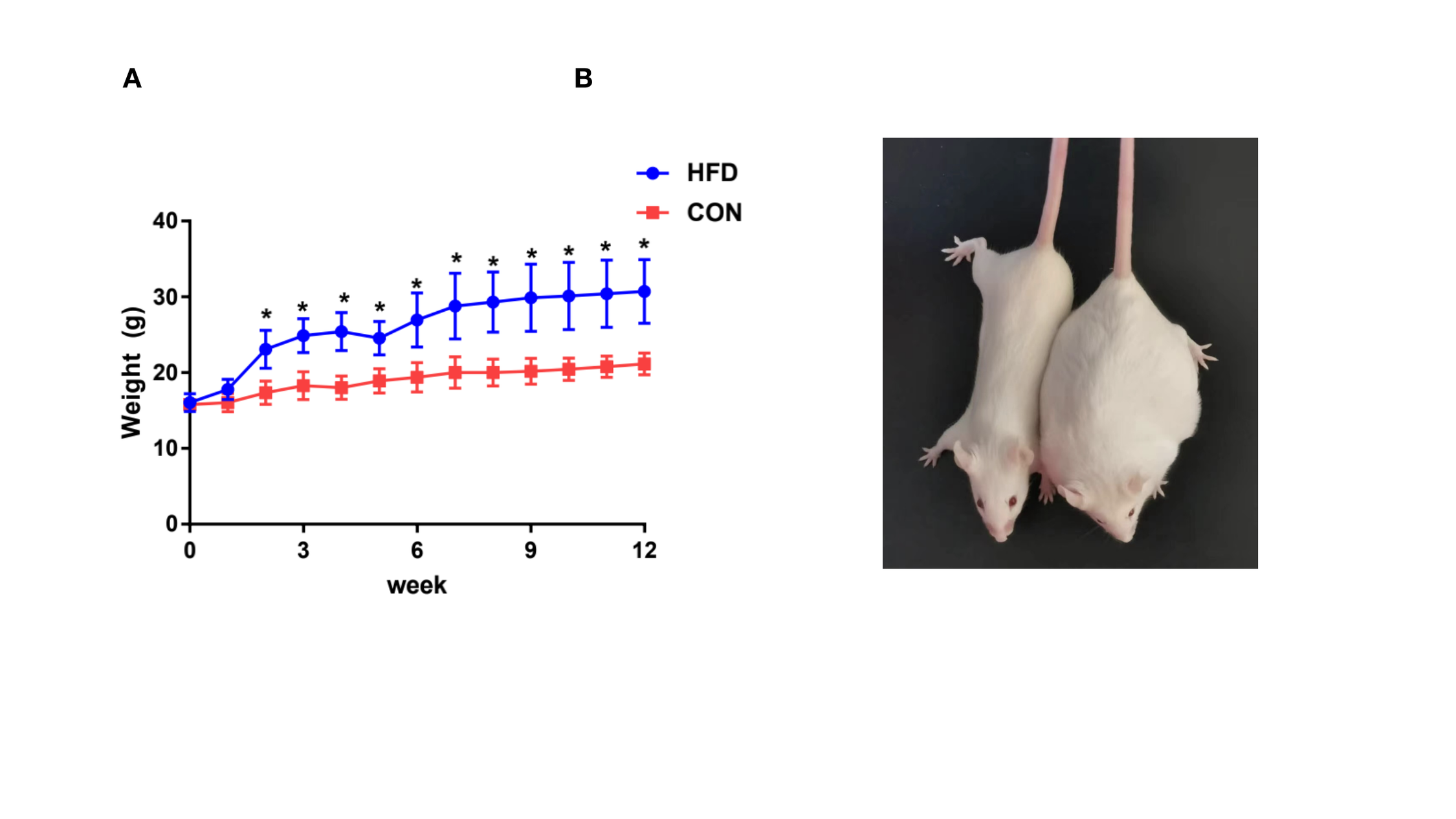

Supplement: S1 Fig — (A) Continuous assessment of the body weights of the mice during the 12-week feeding period. Values are presented as mean + S.E.M. (N = 25), “*” in the figure indicates a significant difference in body weight between the two groups of mice at this time. (B) Comparison of the high-fed diet (HFD) and standard diet (CON) mice at week 12. (TIF) [file pone.0287534.s001.tif]

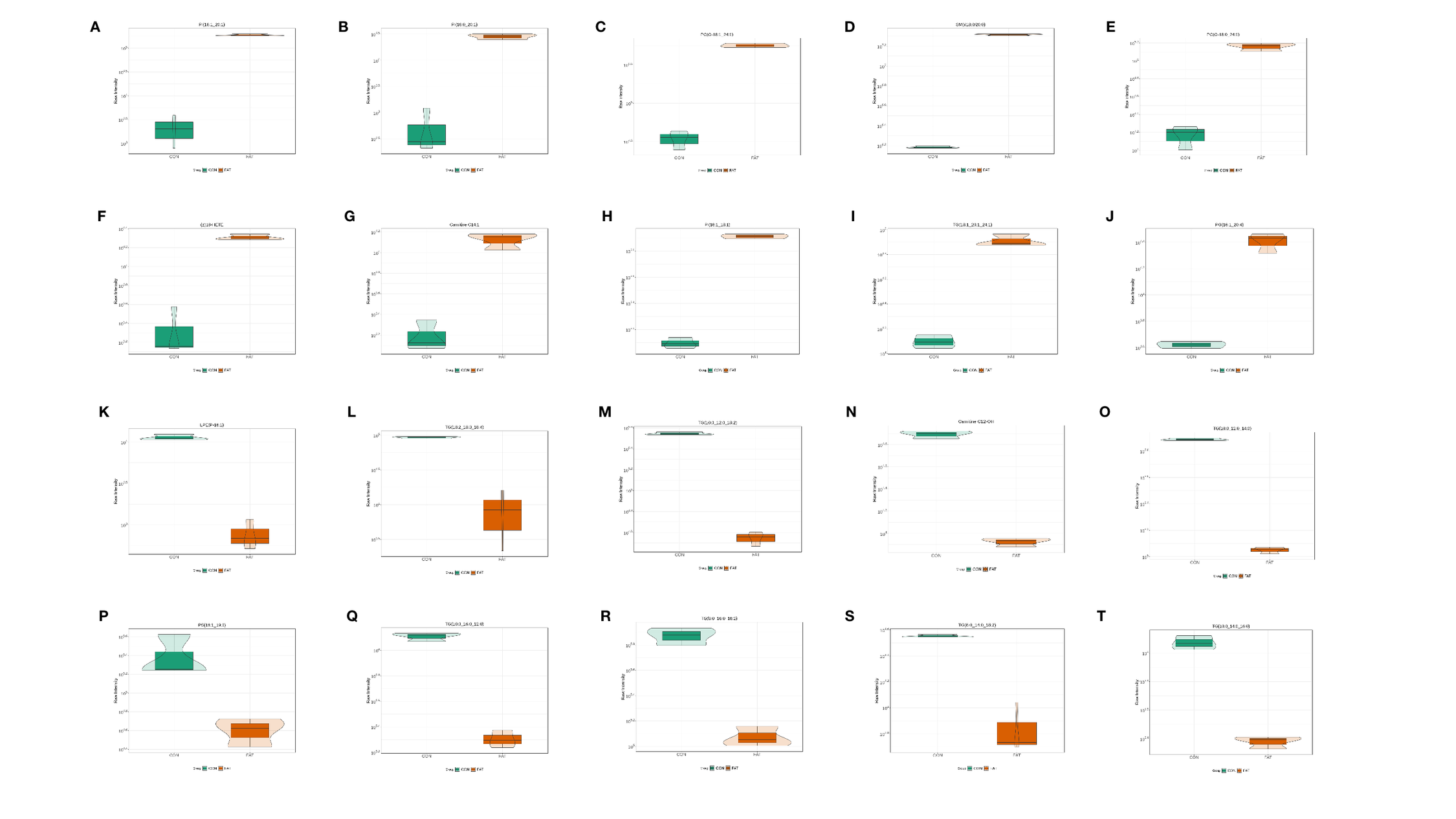

Supplement: S2 Fig — (TIF) [file pone.0287534.s002.tif]
