# Supplementary material for: Effect of high-fat diet on the lipid profile of ovarian granulosa cells and female reproduction in mice
Source: PLoS One. 2023 Jun 27;18(6):e0287534. doi: 10.1371/journal.pone.0287534 (PMC10298767; doi:10.1371/journal.pone.0287534)
Supplement: S1 Table — (PDF) [file pone.0287534.s003.pdf]

Supplementary Table S1 Ingredient composition and nutrient value of the standard feed

Ingredient	Percentage (%)
maize, wheat, alfalfa	60%
fish meal, chicken powder, soybean meal	33%
amino acids, vitamins, minerals	4%
soybean oil, sunflower seed oil	3%
