# Supplementary material for: Effect of high-fat diet on the lipid profile of ovarian granulosa cells and female reproduction in mice
Source: PLoS One. 2023 Jun 27;18(6):e0287534. doi: 10.1371/journal.pone.0287534 (PMC10298767; doi:10.1371/journal.pone.0287534)
Supplement: S2 Table — (PDF) [file pone.0287534.s004.pdf]

Supplementary Table S2 Ingredient composition of high fat diet (HFD)

Ingredient	Percentage (%)
yolk powder	15
sucrose	13
lard	10
casein	5
maltodextrin	1.5
cholesterol	1.2
mineral mix	1
vitamin mix	0.5
cholate	0.2
maintain feed[1]	52.6

[1]maintain feed is the standard feed used for control
